# Bayesian entropy estimators for spike trains

**DOI:** 10.1186/1471-2202-14-S1-P316

**Published:** 2013-07-08

**Authors:** Il Memming Park, Evan Archer, Jonathan Pillow

**Affiliations:** 1 Institute for Neuroscience and Department of Psychology, The University of Texas at Austin, TX 78712, USA; 2 Institute for Computational and Engineering Sciences, The University of Texas at Austin, TX 78712, USA; 3 Division of Statistics & Scientific Computation, The University of Texas at Austin, Austin, TX 78712, USA

## 

Information theoretic quantities have played a central role in neuroscience for quantifying neural codes [1]. Entropy and mutual information can be used to measure the maximum encoding capacity of a neuron, quantify the amount of noise, spatial and temporal functional dependence, learning process, and provide a fundamental limit for neural coding. Unfortunately, estimating entropy or mutual information is notoriously difficult--especially when the number of observations *N *is less than the number of possible symbols *K *[2]. For the neural spike trains, this is often the case due to the combinatorial nature of the symbols: for *n *simultaneously recorded neurons on *m *time bins, the number of possible symbols is *K = *2*^n+m^*. Therefore, the question is how to extrapolate when you may have a severely under-sampled distribution.

Here we describe a couple of recent advances in Bayesian entropy estimation for spike trains. Our approach follows that of Nemenman et al. [2], who formulated a Bayesian entropy estimator using a mixture-of-Dirichlet prior over the space of discrete distributions on *K *bins. We extend this approach to formulate two Bayesian estimators with different strategies to deal with severe under-sampling.

For the first estimator, we design a novel mixture prior over countable distributions using the Pitman-Yor (PY) process [3]. The PY process is useful when the number of parameters is unknown *a priori*, and as a result finds many applications in Bayesian nonparametrics. PY process can model the heavy, power-law distributed tails which often occur in neural data. To reduce the bias of the estimator we analytically derive a set of mixing weights so that the resulting improper prior over entropy is approximately flat. We consider the posterior over entropy given a dataset (which contains some observed number of words but an unknown number of unobserved words), and show that the posterior mean can be efficiently computed via a simple numerical integral.

The second estimator incorporates the prior knowledge about the spike trains. We use a simple Bernoulli process as a parametric model of the spike trains, and use a Dirichlet process to allow arbitrary deviation from the Bernoulli process. Under this model, very sparse spike trains are *a priori *orders of magnitude more likely than those with many spikes. Both estimators are computationally efficient, and statistically consistent. We applied those estimators to spike trains from early visual system to quantify neural coding characteristics.
